# Biomarkers in Prostate-Specific Membrane Antigen Theranostics

**DOI:** 10.3390/diagnostics11061108

**Published:** 2021-06-18

**Authors:** Panagiotis J. Vlachostergios, Ioannis Zachos, Vassilios Tzortzis

**Affiliations:** 1 Department of Medicine, Division of Hematology and Medical Oncology, Weill Cornell Medicine, New York, NY 10065, USA; 2 Department of Urology, Faculty of Medicine, School of Health Sciences, University of Thessaly, University Hospital of Larissa, 41100 Larissa, Greece; johnbzac@yahoo.gr

**Keywords:** prostate specific membrane antigen, theranostics, radionuclide therapies, ^177^Lu, biomarker, predictive, prognostic, PSA response, progression-free survival, overall survival, metastatic castration-resistant prostate cancer

## Abstract

Theranostics of prostate cancer (PC) represents a growing area of development of imaging agents and targeted radionuclide therapeutics against a major target, prostate specific membrane antigen (PSMA). In view of the encouraging efficacy from the use of ^177^Lu and other radionuclides in metastatic castration-resistant prostate cancer (mCRPC), it is becoming increasingly important to identify surrogate markers that can help predict which patients are more likely to respond and experience improved survival. This review discusses potential predictors of efficacy of PSMA-targeted radionuclide therapies (TRT) segregated in three major categories: imaging, clinical and molecular.

## 1. Introduction

Therapy response assessment is a critical step in cancer management, enabling clinicians to optimize the use of therapeutic options during the course of the disease. Prostate-specific membrane antigen (PSMA) is an ideal target for imaging diagnostics and targeted radionuclide therapy (theranostics) of PC and its metastases [1]. Several radionuclides are currently available for the treatment of metastatic PC, such as ^223^Ra, ^177^Lu-PSMA, and ^225^Ac-PSMA. ^177^Lu and ^225^Ac are radionuclides that bind with high affinity to PSMA, which enables β- and α-particle therapy targeted at metastatic castration-resistant prostate cancer (mCRPC), respectively, in both bone and soft-tissue metastases [2].

The agent that is the farthest along in regulatory pathways for approval is ^177^Lu-PSMA-617 [3]. The TheraP randomized phase 2 trial demonstrated a higher response rate, defined as a reduction in the prostate-specific antigen (PSA) level of at least 50%, with ^177^Lu-PSMA-617 compared to third-line cabazitaxel in patients with mCRPC [4,5]. The lower toxicities rate as well as the recent positive findings of the phase 3 VISION trial, which demonstrated a 38% reduction in risk of death (median OS benefit of 4 months) and a 60% reduction in the risk of radiographic disease progression or death (median rPFS benefit of 5 months) from addition of ^177^Lu-PSMA-617 to standard of care compared to best standard of care alone, suggest that it is an active therapy for mCRPC patients [6].

There is currently an unmet need for developing robust biomarkers to inform treatment decisions and identify patients who are likely to respond. Validated biomarkers that capture the complexity of the biology of the target within the tumor as well as the tumor microenvironment are lacking. This review will focus on currently studied indicators of efficacy of PSMA-targeted radionuclide therapies (TRT) segregated in three major categories: imaging, clinical and molecular. A comprehensive computer literature search of PubMed/MEDLINE database was performed by two authors independently to find relevant published articles on biomarkers in PSMA theranostics for mCRPC. A search algorithm based on a combination of the following terms was used: (A) “PSMA” AND (B) “theranostics” OR “radionuclide” AND C) “prostate” OR “prostatic” AND D) “predictive” OR “response” OR “prognostic” OR “survival”. No beginning date limit nor language restrictions were used. The last update of the literature search was 14 June 2021. To expand the search, references of the retrieved articles were also screened, searching for additional studies.

## 2. Imaging Biomarkers

### 2.1. PSMA Uptake

#### 2.1.1. PSMA Total Tumor Volume

The total volume of metastatic disease on PSMA imaging was studied with respect to prognosis of patients with mCRPC. Total tumor volume (TTV) can be calculated by summing the volumes of segmented lesions to obtain the whole-body tumor volume, after subtracting physiologic PSMA accumulation to the liver, spleen, bladder, kidneys, small bowel, tear and salivary glands) from foci with pathologic PSMA uptake [7]. In a cohort of 40 patients treated ^177^Lu-PSMA, the semiautomatically quantified tumor volume (PSMATV50) was significantly associated with overall survival (OS) of these patients, independently of other important prognostic factors including alkaline phosphatase (ALP) and prostate-specific antigen (PSA) [7].

In another study, TTV was derived using a threshold-based volume of interest (VOI) extracted from the complete field of view (FoV). The lower threshold was defined as the mean standard uptake value (SUV) derived from a cubic 10 × 10 × 10 voxel reference VOI of the liver plus 20% to avoid most of the nonspecific and physiological PSMA 11 uptake [8]. The reference VOI was manually drawn by a single investigator avoiding the inclusion of major intrahepatic vessels based on computed tomography (CT)/magnetic resonance imaging (MRI). A program-inherent segmentation algorithm was then applied to the threshold derived VOI, enabling the deletion of the most common significant noncancer uptake areas (kidney, salivary glands, gut, spleen, bladder). Nonspecific uptake still present was then cropped manually [8]. Among 38 patients who underwent ^177^Lu-PSMA-617 radioligand therapy (RLT), change in TTV pre- and post-RLT was significantly associated with PSA response and OS, after a median follow-up of 17 months [8].

In a more stringent imaging analysis of 50 patients from the phase 2 Lu-PSMA trial using both fluorodeoxyglucose (FDG)- and PSMA-positron emission tomography (PET), patients with low volumes of FDG avid disease had a longer OS than other patients whereas the volume of tumor burden on PSMA PET/CT was not prognostic [9]. As an explanation, the authors of this study suggested that in this setting of PSMA-avid advanced PC, the volume of aggressive disease defined by FDG may have a much higher impact on patient outcome compared to the volume of PSMA-avid disease which is effectively targeted by PSMA-RLT [9].

An alternative way of semiautomatic calculation of PSMA TTV involves the use of a volume measurement software named METAVOL, which was initially developed for FDG-PET to measure metabolic tumor volume (MTV) as well as SUV_max_ or SUV_mean_ [10]. Similar to MTV, PSMA tumor volume (PSMA-TV) as well as SUV_mean_ of individual lesions is automatically provided by METAVOL after VOI determination. Whole body PSMA-TV (wbPSMA-TV) of each patient is equal to the sum of PSMA-TV of all lesions. Total lesion PSMA uptake (TL-PSMA) is obtained by multiplying the PSMA-TV and SUV_mean_ of each lesion. Whole body TL-PSMA (wbTL-PSMA) of each patient is equal to the sum of TL-PSMA of all lesions [10]. While wbPSMA-TV and wbTL-PSMA were significant predictors of progression-free survival (PFS) in this heterogenous cohort of newly diagnosed PC patients, with only 30 being metastatic and none treated with PSMA-RLT, their value in monitoring treatment and predicting OS specifically after PSMA-RLT remains to be elucidated [10].

Collectively, despite methodological differences in measuring PSMA TTV, it appears to be a promising surrogate for response to PSMA-RLT and for OS.

#### 2.1.2. PSMA Tumor Intensity

Tumor PSMA intensity represents a metric that could be used as an alternative or complementary to PSMA TTV to predict responses and clinical outcomes after treatment with PSMA-RLT. As with PSMA TTV, the exact definition of various scales of PSMA uptake differs between studies.

One group has used a five-point imaging score (IS) which was assigned based upon PSMA uptake in tumors compared to liver uptake and scored by two independent radiologists on a 0–4 scale [11,12]. PET images were scored by averaging SUV_max_ of the five lesions with highest uptake and then comparing that value with liver SUV_mean_. Planar single photon imaging was also studied, scoring the three lesions with the highest uptake. Using this assessment in 215 men with progressive mCRPC who were treated with β-emitting radionuclides (^177^Lu-J591, ^177^Lu-PSMA-617, ^90^Y-J591, ^177^Lu-J591 + ^177^Lu-PSMA-617), a high IS of 2–4 was independently associated with ≥50% decrease in PSA, after accounting for CALGB (Halabi) prognostic score, dose administered, and previous taxane use [11].

^68^Ga-PSMA-11 SUV has been associated with PSA reduction in other studies, as well [13]. Using a detection threshold of SUV > 3, the mean intensity of PSMA-avid tumor uptake correlated with OS in men with mCRPC treated with ^177^Lu-PSMA-617 [9,14]. In these studies, PSMA intensity at sites of disease had to be significantly greater than that in normal liver, as defined by a tumor SUV_max_ at least 1.5 times the SUV_mean_ of liver. Patients were excluded if ^18^F-FDG PET demonstrated discordances including sites of ^18^F-FDG–positive and PSMA-negative disease, which the investigators anticipated would be less likely to respond to therapy [14].

A more simplified approach dichotomizing PSMA uptake into intense (>salivary gland uptake) and low (<salivary gland uptake) was able to predict responders regardless of neuroendocrine differentiation status evidenced by serum and immunohistochemical neuroendocrine markers [15]. Overall, PSMA tumor intensity could become a useful tool to select the population of patients more likely to benefit from PSMA-TRT.

### 2.2. Fluorodeoxyglucose (FDG) Uptake

The clinical utility of tumor burden assessment with FDG as a radiotracer using PET has been extensively studied in several types of cancer, including PC. In general, various imaging assessment tools including SUV_max_ of the hottest lesion, total metabolic tumor volume (MTV), and total lesion glycolysis (TLG) have been tested for their prognostic value in patients receiving systemic therapies including taxanes and androgen receptor targeted agents, with MTV and TLG being the most robust [16]. The sum of SUV_max_ derived from ^18^F-FDG PET/CT contributes independent prognostic information on OS of mCRPC patients even after adjusting for relevant clinical parameters, including serum PSA level, ALP, use of pain medication, prior chemotherapy, and Gleason score at initial diagnosis [17]. Analysis of the therapeutic response to Lu-PSMA in 35 patients with FDG-PET showed that high FDG uptake (SUV_max_ > 15) correlated with a high Gleason score >8, and lack of response, progressive disease and short PFS [18].

When combined with PSMA-PET in patients receiving PSMA-TRT, a high FDG-positive tumor volume defined as SUV > liver+2sd predicted shorter survival in men undergoing Lu-PSMA therapy [9]. Patients treated with Lu-PSMA who had low PSMA expression and discordant FDG avid disease had also poor OS, with a median of 2.5 months [19]. Hence, addition of FDG PET/CT to identify discordant disease appears to assist in optimal selection of patients most likely to benefit from PSMA-TRT. In another study, retrospective comparison of patients with at least one FDG-positive, but PSMA-negative (FDG+/PSMA-) lesion to patients without any FDG+/PSMA- lesions revealed a significant lower OS in the latter group, providing corroborating evidence that FDG+/PSMA- lesions are a negative predictor of OS in patients with mCRPC undergoing RLT [20].

## 3. Clinical Biomarkers

### 3.1. Early PSA Changes

Serum PSA represents a valid surrogate for assesment of response to chemotherapy and androgen directed therapies in mCRPC. The Prostate Cancer Clinical Trials Working Group 3 (PCWG3) recommendations have retained the criterion of 12 weeks for defintive assessment of 30% and 50% responses [21]. Based on that, many PSMA-TRT studies have also shown improved outcomes in this setting, for example patients achieving PSA decline of ≥50% within 12 weeks of treatment showed longer clinical PFS and OS [22].

Being able to recognize an early signal of response could be of clinical value. There is scarce evidence that early decline of PSA within 12 weeks after the administration of ^177^Lu-PSMA may improve the oncological outcomes in patients with mCRPC. Patients with mCRPC having >21% PSA decline after one cycle of ^177^Lu-PSMA and prior to receiving the second cycle, thus in less than 8 weeks, experienced longer OS compared to those who did not reach that early cut-off [23]. Any initial PSA decline after the first cycle of ^177^Lu-PSMA-617 correlated with prolonged OS (15.5 vs. 5.7 months) in another cohort of 109 mCRPC patients [24].

A comprehensive comparative analysis of early PSA assessment at 6 weeks after receiving ^177^Lu-PSMA therapy in 124 mCRPC patients demonstrated that a decline ≥ 30% predicted a longer OS compared to PSA stability or progression, and was further associated with a lower risk of radiographic progression, suggesting that early PSA decline could serve as a very early decision tool to continue or switch treatment [25]. One caveat that should be taken into consideration is the likelihood of early PSA flare which is however rare with PSMA-TRT (1%), unlike other types of systemic therapy, such as taxanes [25].

### 3.2. PSA Doubling Time (PSADT)

PSADT, calculated using the three most recent PSA values (ng/dL) in chronological order [26] is a strong predictor of metastases, all-cause mortality, and PC-specific mortality in men with non-metastatic CRPC [27]. This association is less clear in men with mCRPC undergoing PSMA-TRT.

In a cohort of 40 mCRPC patients who received at least two cycles of ^177^Lu-PSMA-617 PRLT, those with negative PSADT experienced superior one-year PFS as compared to those with positive serum PSA-DT (52.5% vs. 47.5%) [18]. Other retrospective studies did not confirm a predictive or prognostic role of PSADT. Pre-treatment PSADT calculated using PSA levels prior to the first cycle of RLT with cut-off <3 months was not associated with PFS or OS in 59 patients with mCRPC treated with Lu-PSMA after failure of novel androgen signaling pathway inhibitors and chemotherapy [28]. In a prospective phase 2 study of ^177^Lu-PSMA-617, baseline PSADT was not predictive of OS [9]. These findings could possibly reflect the negative impact of a larger disease burden on the efficacy of PSMA-TRT.

### 3.3. Lactate Dehydrogenase (LDH)

LDH is a cytosolic enzyme released in serum in relation with cell turnover and can reflect disease burden in mCRPC, particularly that of liver metastases [29]. The presence of elevated LDH, particularly the rise by ≥50 U/L was an independent predictor of shorter clinical PFS and OS in 100 mCRPC patients who received 1–6 cycles of ^177^Lu–labeled PSMA imaging and therapy (I&T) [22]. Elevated baseline LDH prior to PSMA-RLT demonstrated a strong prognostic value and was also associated with increased risk for progression under PSMA-RLT [30]. The utility of a “kinetics” approach measuring both baseline and follow-up (after 2–3 months) levels of LDH was also demonstrated in a retrospective analysis that included 137 patients receiving Lu-PSMA [31]. Both elevated LDH at baseline (cut-off: 248 U/L) and stable or decreased values post-PSMA-RLT significantly correlated with OS, independently of other markers, including ALP, PSA, and pro-gastrin-releasing peptide (pro-GRP) [31].

### 3.4. Neuroendocrine Serum Markers

Chromogranin A (CgA) is an acidic glycoprotein usually expressed in neuroendocrine cells, include PC with neuroendocrine differentiation [32]. As this state is not uncommon in heavily pretreated mCRPC patients, studies and metaanalyses addressed the question of its potential predictive and prognostic value. In mCRPC patients treated with abiraterone, serum CgA level more than three times the upper limit of normal predicted PFS and showed a trend towards OS prediction [32]. In either the first or second-line systemic therapy setting, a high CgA level has a negative influence on OS and PFS, and a rising CgA translates in shorter PFS [33].

There is limited evidence to support such a role of CgA in mCRPC patients treated with PSMA-TRT. Increased CgA level in 100 patients receiving ^177^Lu-PSMA treatment had a moderate impact as a negative prognostic marker in general but was specifically related to the presence of liver metastases [30]. Testing a broader panel of neuroendocrine serum markers including CgA, pro-GRP and neuron-specific enolase (NSE), in 50 mCRPC patients undergoing ^177^Lu-PSMA-617 RLT failed to reveal any associations with treatment failure or early progression [15].

Taken together, these findings, along with known inherent limitations of nonspecific increase of CgA due to numerous confounders such as proton pump inhibitors, gastritis or renal insufficiency in more than one third of patients, render these neuroendocrine markers rather not helpful in prediction of PSMA-TRT-related outcomes. One exception to this could be the use of NSE as a potential laboratory indicator for [^18^F]-FDG/[^68^Ga]Ga-PSMA-11 mismatch findings as those lesions are not affected by PSMA-RLT and a change in therapy management is needed [34].

### 3.5. Hemoglobin (Hb)

Anemia is common among patients with mCRPC and has prognostic relevance, with several factors impacting on its magnitude, including the disease itself, particularly in presence of bone marrow infiltration and systemic therapies [35,36]. A retrospective analysis of 61 patients with mCRPC treated with ^177^Lu-PSMA-617 demonstrated that a normal pre-treatment Hb level was predictive of ≥50% PSA decline 4 weeks after receiving the third PSMA-RLT dose as well as of OS compared to patients with reduced baseline Hb [37]. Likewise, another group also reported that lower pretreatment levels of Hb are univariately associated with lack of PSA decline [38].

### 3.6. Platelet (PLT) Count

There are scarce data regarding the clinical utility of PLT count in patients treated with PSMA-TRT. In a comprehensive analysis of various clinical predictors of response in 40 progressive mCRPC patients receiving ^177^Lu-PSMA, a platelet count of more than 300 had an independent negative effect on therapeutic response in these patients, regardless of other clinical parameters including age, γ-glutamyl transferase, LDH, hemoglobin, Gleason score, C-reactive protein, and regular need for pain medication [38].

### 3.7. Alkaline Phosphatsase (ALP)

The value of ALP in predicting OS is well established in mCRPC patients undergoing first-line chemotherapy [36]. With respect to PSMA-RLT, retrospective analyses reported a combined predictive and prognostic role of initial ALP level <220 in terms of PSA PFS (41 vs. 18 weeks) and OS (56 vs. 28 weeks), respectively [28]. A stricter cut-off of <120 and stable and/or decreased values of ALP post-PSMA RLT were also reported to effectively identify patients who will experience longer survival [31].

### 3.8. Pain Medication

The regular use of opiate analgesics in patients with mCRPC is a surrogate indicator of poor OS [36]. The α-emitter ^223^Ra was able to improve OS in all mCRPC patients with symptomatic bone metastases enrolled in the phase 3 ALSYMPCA trial, regardless of opioid use [39]. The prognosis of mCRPC patients with and without opiate analgesic requirements is also important for clinicians administering PSMA-RLT. Retrospective analysis of 40 hormone-refractory patients with distant metastases and progressive disease treated with ^177^Lu-PSMA revealed that the regular need for analgesics had a negative impact on PSA response (>50% or any PSA decline) [38]. Accordingly, regular need for analgesics showed a worse PSA response in 52 men undergoing multiple cycles of RLT with Lu-PSMA-617 [40] and was a negative predictor of OS in a separate study of the same group [41].

### 3.9. Visceral Metastases and Extent of Osseous Metastases

The presence of visceral metastases is a harbinger of poor OS in patients with mCRPC undergoing systemic chemotherapy [36]. It appears that there is a similar impact on those receiving PSMA-TRT. Various groups reported poor PSA response and shorter clinical PFS or/and OS in those patients with visceral disease who were treated with ^177^Lu-PSMA-617 [22,41]. Further, a systematic review and metaanalysis of 12 studies comprising 1504 patients in total, suggests that presence of visceral metastases not only predicted low biochemical response rate but was also a significant prognosticator of worse PFS and OS [42]. Interestingly, the site of metastasis matters, as patients with or without lung metastases did not differ in OS whereas patients with liver metastases had a worse OS than patients without liver metastases. Patients with lymph node-only metastases or/and one or two bone lesions had the best OS. [24,43]. This could reflect changes in tumor biology during the progression of metastatic PC and is also in line with another study that stratified the prognostic role of bone disease burden in four groups with distinct OS (18 months in patients with <6 lesions, as opposed to 13 months for 6–20 lesions, 11 months for > 20 lesions and only 8 months in patients with diffuse osseous involvement, respectively). Thus, the greater the extent of bone involvement the poorer the OS after RLT [44].

### 3.10. Composite Predictive Score

Given the presence of multiple imaging and clinical factors that can influence therapeutic benefit and OS after PSMA-TRT, an effort was made to retrospectively assess the predictive value of key markers such as ALP (cut-off 135 U/L), PSA (cut-off 200 ng/mL) and SUV_max_ of the “hottest lesion” in pre-therapeutic PET in a composite predictive score [45]. This exploratory analysis was conducted in 46 patients who received two cycles of ^177^Lu-PSMA. The composite score separated two distinct groups of patients: one with ≤2 predictive factors that demonstrated only a 19% risk of progression, and another with three predictive factors, resulting in 90% chance of progressive disease [45]. Prospective validation of these findings is warranted.

## 4. Molecular Biomarkers

### 4.1. PSMA in Tumor Tissue

Retrospective tumor microarray studies of PSMA expression in primary prostate tumor tissues supported its prognostic utility as a potential indicator of lethal disease, correlating with a higher Gleason score and PSA at diagnosis [46]. High expression of the FOLH1 gene encoding PSMA assessed in radical prostatectomies via use of the Decipher test also correlates with high Gleason grade, high androgen receptor activity scores, and the luminal subtype [47]. PSMA expression is higher in lymph node metastases compared to primary tumors and has been associated with a shorter time to biochemical recurrence after radical prostatectomy [48]. Likewise, high PSMA on prostate biopsies predicts disease recurrence following curative therapy [49].

The question of whether PSMA overexpression in mCRPC patients undergoing treatment with PSMA-TRT could represent a prognostic or predictive tool has yet to be fully elucidated. In a small cohort of 13 patients who received targeted alpha radiation therapy with ^225^Ac-labeled PSMA ligands, those with high baseline immunohistochemical PSMA expression H-score of ≥200, defined semiquantitatively on a scale of 0−300 as a composite of the percentage of immunopositive tumor cells (0−100%) and the staining intensity (0 = negative; 1+ = weak; 2+ = moderate; 3+ = intense), tended to have longer OS compared to those with H-score < 200 (12.8 vs. 1.8 months, respectively) [50].

An issue with semiquantitative immunohistochemical assessment of membranous PSMA is the marked tumor heterogeneity among patients and among different metastases within the same patient [51]. For example, out of 38 men with castration-sensitive PC, 42% had no detectable expression of membranous PSMA on diagnostic biopsies (H-score < 10) [51]. Further, although there seems to be consistency on the association of membranous PSMA with a more aggressive histological phenotype and poor prognosis [51], drawing definitive conclusions on the impact of PSMA expression on survival is challenging, given the small size of individual studies. Additionally, different cut-offs used across studies (e.g., 100 vs. 17.5) hamper a clearer interpretation and segregation between positive-negative and high-low states of PSMA expression.

### 4.2. PSMA in Circulating Tumor Cells (CTCs)

During progression to the metastatic, castration-resistant state, the median PSA PFS and OS are significantly shorter in patients with PSMA-positive CTCs compared to those without PSMA mRNA expression [52]. Additionally, PSMA transcript level is overall a surrogate indicator of poor response to currently approved treatments for mCRPC, including enzalutamide, abiraterone, docetaxel, and cabazitaxel [52]. This is not necessarily the case for patients treated with ^177^Lu-PSMA-617 RLT, according to a small cohort of 19 men with mCRPC [53]. Although PSMA mRNA expression in CTCs correlated with baseline serum PSA and with the MTV on PSMA PET/CT, there was no association with PFS or OS following treatment with ^177^Lu-PSMA-617 [53].

### 4.3. DNA Damage Response and Repair (DDR) Gene Alterations

A main cause of tumor heterogeneity resulting in target heterogeneity (PSMA), is the presence of genomic instability. Genomic instability is often associated with a defective DNA repair machinery. On the other hand, DNA repair defects may shape the response of tumors to DNA damaging agents, including ionizing radiation. Therefore, assessment of DDR gene alterations could be instrumental in predicting response or resistance to PSMA-TRT. This hypothesis was tested by a few studies. Using targeted next-generation sequencing analysis of tumor biopsies and germline DNA samples from mCRPC patients one study found that presence of deleterious DDR mutations in BRCA2, ATM, mismatch repair (MMR), and CDK12 genes was more frequent in patients with high tumor membranous PSMA expression [51]. Transcriptomic analysis in another mCRPC cohort revealed an inverse relationship between membranous PSMA expression (high) and BRCA2 (loss) as well as double strand break repair activity (low expression of mRNA signature), suggesting that DNA repair status could represent a mirror image of PSMA expression in mCRPC [51].

The presence of pathogenic BRCA1 mutations was associated with longer OS (16.1 vs. 7.6 months) after treatment with ^25^Ac-PSMA-617 [50]. Anecdotal response of ATM mutation-positive mCRPC to beta-radiation with ^177^Lu-PSMA-617 and concomitant enzalutamide is in line with a potential role of DDR defects in sensitizing to PSMA-TRT [54]. DDR alterations are also present in non-responders to ^225^Ac-PSMA-617 [55]. However, since the majority of this small patient series (4/7) had already progressed after prior PSMA-TRT it is less clear that pathogenic DDR alterations were truly driving this resistant phenotype. Moreover, tumors from heavily pre-treated patients transitioning to a neuroendocrine phenotype driven by TP53- and RB1- loss, are characterized by elevated DNA repair gene expression signature scores and PSMA suppression [56,57].

In the largest to-date single-institution cohorts of mCRPC patients treated with beta- or/and alpha-emitting PSMA-TRT, deleterious somatic BRCA1 or BRCA2 alterations (copy number variations and point somatic mutations) were found to correlate with longer PFS and PFS/OS respectively, whereas TP53 alterations predicted shorter PFS [58]. The presence of germline or somatic BRCA2 alterations (inactivating mutations, deletions or losses) within a broad panel of DDR genes was also associated with PSA response and emerged as an independent prognostic indicator of OS after adjusting for CALGB (Halabi) prognostic groups [59].

### 4.4. Neurotensin Receptor 1

A particular challenge for the efficacy of PSMA-TRT, discussed earlier, is attenuated expression of PSMA during development of resistance or as a manifestation of tumor heterogeneity. To counteract this limitation from an otherwise good but imperfect biomarker (PSMA), additional molecular targets for patient screening, detection of metastatic disease and treatment monitoring are under investigation. One such target is neurotensin receptor1 (NTSR1), which exhibits high or moderate expression in 92% of PC tumors, including all PSMA-negative tissues, suggesting a potential complementary role in targeted imaging or/and therapy [60]. Although the value of NTSR1 expression was not assessed in patients with PSMA-negative PET/CT, preclinical in vitro and in vivo studies in PC animal models suggest that PET imaging using specific probes for NTSR1, such as ^68^Ga-DOTA-NT-20.3 or ^18^F-DEG-VS-NT could represent viable alternative options for selecting therapies targeting NTSR1 in mCRPC patients with limited PSMA expression levels [60,61].

## 5. Conclusions and Perspectives

In a world where precision medicine is becoming the standard approach in oncology, PSMA PET has the potential of being the best available imaging method for mCRPC therapy response evaluation. Along with it, other key indicators at the clinical or/and molecular level, may be able to guide treatment decisions regarding PSMA-TRT (Table 1).

Since the majority of biomarker signals are derived from retrospective studies, it will be critical to conduct prospective PSMA-TRT trials with a biomarker-embedded design rather than as exploratory variables in order to validate the true significance of each one. Additionally, because different cut-off values have been reported for several of the studied markers, harmonization of data is required for future dichotomous analyses. The phase III VISION study was recently reported as a positive trial, demonstrating the superiority of addition of ^177^Lu-PSMA-617 to standard of care in terms of radiographic PFS (8.7 vs. 3.4 months) and OS (15.3 vs. 11.3 months) in men with mCRPC [6]. It will be important to analyze whether any of the described parameters could have impacted these results.

The predictive and prognostic utility of DDR defects could assist with patient selection and could further be exploited to optimize the activity of PSMA-TRT by adding PARP inhibitors, platinum agents, immune checkpoint inhibitors or other pharmacologic agents targeting DNA repair.

One limitation in establishing surrogates of response to PSMA-TRT is that there are specific effects of other systemic therapies, particularly androgen deprivation therapy on PSMA expression by PC cells, leading to potential pitfalls and dynamic changes in the actual target (PSMA) [62,63,64].

Consequently, it is less likely that one single biomarker would be sufficient to accurately predict responses and outcomes after treatment with PSMA-TRT. Instead, a combination of biomarkers with strong biological rationale each being able to provide complementary predictive and prognostic value would be more likely to accompany the future integration of PSMA-TRT in the armamentarium of systemic therapies for mCRPC.

## Figures and Tables

**Table 1:** Putative predictive and prognostic indicators of outcomes after PSMA-TRT in mCRPC.

Biomarker	Predictive	Prognostic	Both
PSMA TTV			●
PSMA intensity			●
FDG SUVmax			●
PSA change in <12 wks			●
PSADT	●		
LDH			●
NE markers (CgA, NSE, pro-GRP)		●	
Hb			●
PLT	●		
ALP			●
Pain medication			●
Visceral metastases (liver)			●
Composite predictive score	●		
PSMA in tumor		●	
DDR (BRCA1, BRCA2, ATM, TP53)			●
